# Evaluating the feasibility and acceptability of self-injection of subcutaneous depot medroxyprogesterone acetate (DMPA) in Senegal: a prospective cohort study^☆^^☆☆^^★^

**DOI:** 10.1016/j.contraception.2017.06.010

**Published:** 2017-09

**Authors:** Jane Cover, Maymouna Ba, Jeanette Lim, Jennifer Kidwell Drake, Bocar M. Daff

**Affiliations:** a PATH, PO Box 900922, Seattle, WA 98109, USA; b PATH, BP 15115, Dakar-Fann, Dakar, Senegal; c Senegal Ministry of Health and Social Action, DSRSE, VDN, Cite Keur Gorgui, BP 4024, Dakar, Dakar, Senegal

**Keywords:** Home and self-injection, Self-administration, DMPA-SC, Sayana® Press, Injectable contraception, Family planning

## Abstract

**Objectives:**

Expanding contraceptive options through self-injection may improve access and confidentiality. There are few published studies on contraceptive self-injection in sub-Saharan Africa and none in West Africa, a region with high unmet need. This study was performed to assess feasibility of subcutaneous DMPA self-injection in Senegal; objectives were to (1) measure the proportion of participants who self-injected competently 3 months after training, (2) measure the proportion who self-injected on time (defined conservatively as within 7 days of reinjection date), and (3) assess acceptability of self-injection.

**Study design:**

In this prospective cohort study, 378 women aged 18–49 years were trained to self-inject by study nurses. Three months later, women returned unprompted to the clinic to self-inject, and technique and visit timing were evaluated. Women continuing with a third self-injection were followed up at home after their next scheduled injection date. At each interaction, participants were interviewed to learn about their experience; additional questions during the final home visit focused on storage and disposal practices, and acceptability.

**Results:**

Among the 337 participants followed up 3 months post-training, 310 self-injected, and 87% did so competently. Factoring in women who declined to self-inject, electing to have the provider administer the injection instead, a total of 80% [95% confidence interval (CI) = 75–84%] self-injected competently 3 months post-training, and 84% [95% CI=80–88%] reinjected on time, while 72% [95% CI=67–77%] were both on time and competent. The vast majority (93%) expressed a desire to continue.

**Conclusions:**

Self-injection is feasible and acceptable among most study participants in Senegal.

**Implications:**

These first research results on contraceptive self-injection in West Africa indicate initial feasibility and acceptability of the practice. Results underscore the importance of designing self-injection programs that empower and support women, including those with limited education.

## 1 Introduction

Senegal is one of many countries in sub-Saharan Africa with high unmet need for family planning (25%) among married women [1]. Injectable contraception is the most popular method in Senegal [1]. Expanding service-delivery options through self-injection may improve injectable access — particularly in remote areas — by eliminating the need to return quarterly to a clinic for reinjection. Self-injection may also enhance confidentiality for women who need to leave the home to obtain contraceptives without the knowledge of spouses or other family members.

Subcutaneous depot medroxyprogesterone acetate (DMPA) or DMPA-SC is a variation of intramuscular DMPA (DMPA-IM) that contains 104 mg rather than 150 mg of DMPA, administered as a subcutaneous injection. DMPA is effective and safe in either form. A review of 14 studies concluded that DMPA-SC and DMPA-IM are therapeutically equivalent, with similar side-effects profiles [2]. Aggregated data from seven clinical trials of DMPA-IM and two trials of DMPA-SC revealed a first-year failure rate of 2 per 1000 women [3].

The DMPA-SC product Sayana® Press is manufactured by Pfizer Inc. and packaged in the Uniject™ injection system. PATH designed and developed Uniject™ with collaboration from Horizon Medical, Inc. In 1996, PATH licensed the Uniject™ device technology and related patents for commercialization to Becton, Dickinson and Company (BD). PATH also helped connect BD with the DMPA manufacturer, which ultimately led to Pfizer Inc.’s development of the product now marketed as Sayana® Press. Sayana® Press received regulatory approval in Europe in 2012 and Senegal in 2014. Acceptability studies found that most injectable clients and providers in Senegal preferred this DMPA-SC product over DMPA-IM [4], [5]. In 2015, the UK Medicines and Healthcare products Regulatory Agency (MHRA) approved relabeling Sayana® Press to support self-injection [6] and the World Health Organization (WHO) now recommends self-injection for women who receive appropriate information, training, and support [7]. The process for pursuing relabeling for self-injection in several developing countries, including Senegal, was initiated in 2016.

The studies published on contraceptive self-injection have found that, overall, women found the practice convenient and easy, and there were no pregnancies among women practicing self-injection in Pfizer Inc.’s original clinical trials of DMPA-SC delivered in a prefilled syringe [8] or in studies in Florida [9], Scotland [10], and New York [11]. That said, these studies involved limited women-months of exposure due to small samples and short timeframes. A clinic-based study in Brazil of self-injection of a monthly injectable also using the Uniject™ delivery system, found high levels of competence but also that nearly half of the women invited to self-inject opted not to do so [12]. In a study of DMPA-SC self-injection in Uganda, 87% of women who reinjected after 3 months were both competent and on time [13]. Following on the heels of the Uganda study, the current study is the second to focus on contraceptive self-injection in sub-Saharan Africa. Self-injection studies are underway in Malawi and the Democratic Republic of Congo.

The purpose of this study was to assess feasibility and acceptability of self-injection with DMPA-SC in Senegal. The study's primary objectives were to measure the proportion of participants who demonstrate competent self-injection technique 3 months after training, and to measure the proportion who self-inject on time. The secondary objective was to assess the acceptability of self-injection, expressed as the desire to continue with self-injection and likelihood of recommending the practice to others.

## 2 Material and methods

### 2.1 Study design and procedures

This prospective cohort study was conducted in eight public facilities in two regions of Senegal from September 2015 to July 2016: three periurban health centers in the Dakar region, and three health posts and two peripheral health huts in the Thiès region. Women aged 18–49 years were asked to provide written informed consent to undertake self-injection and participate in interviews. Participants were family planning clients who had chosen injectable contraception and met the standard eligibility criteria for injectable use, as per Ministry of Health guidelines. Women who did not permanently reside in the area, felt unwell on the day of enrollment, or did not speak either French or Wolof were excluded from the study. The study was approved by the Senegal National Health Research Ethics Committee (CNERS) and the PATH Research Ethics Committee.

Study procedures were implemented by licensed nurses and midwives who were trained in DMPA-SC administration, research ethics (including Good Clinical Practice), interviewing, data management, and counseling women for self-injection.

Study staff trained participants one-on-one to do self-injection, guided by a client instruction booklet (Appendix A) [14]. Instruction booklets were designed for low-literacy audiences and pretested in Senegal to maximize usability. Participants practiced the injection on a prosthetic device as many times as needed, using the booklet for guidance, until they achieved competence (study definition of competence detailed in Section 2.2). Training topics included injection technique, calculation of injection dates, review of DMPA side effects, HIV prevention, safe home storage, and disposal practices (e.g., placing the used device in an impermeable container before disposing of it in a latrine or retaining the device until giving it to a health worker).

Immediately after training, women self-injected under supervision of study staff. Their self-injection technique was evaluated using an observation checklist (Appendix B) [15]. In the event that the study nurse felt that an injection was so poorly done as to risk reduced contraceptive efficacy, she could provide a 2nd injection. At this visit, structured baseline and post-injection interviews were conducted.

Three months later, participants who wished to continue self-injection (regardless of whether competent at the previous visit) returned to the clinic to self-inject again while study staff evaluated the woman's technique. No additional training or guidance was provided at the second clinic visit prior to self-injecting, since the intent was to assess whether women would recall the injection technique without additional instruction (as if she were unsupervised at home). Participants seeking guidance were reminded to refer to the client instruction booklet. If a woman had not returned to the clinic when due for her reinjection, she was contacted after 7 days had passed since her scheduled injection date. Women not having returned prior to the contact were considered to be late.^i^ If the woman chose not to self-inject but wished to continue with the method, the nurse administered the injection.

In interviews conducted at the second visit, women were queried about their experience with side effects (including injection site reactions) since their previous injection, their self-injection experience, and their desire to continue (or not) with self-injection.

Women continuing with self-injection were then given one DMPA-SC unit, client instruction booklet, and reinjection calendar with the reinjection date marked to take home. Participants were instructed to self-inject independently at home when due for their next injection.

Home visits were conducted at least 1 week after the next scheduled injection (the third injection). At the follow-up home visit, the study nurse interviewed each woman about her injection experience, the timing of the reinjection, and any injection-site reactions she experienced. If she forgot to give the injection, she could do so at that time, but women who had not reinjected prior to the home visit were considered late. If she decided not to give herself the injection, the study nurse gave the injection or referred her to the clinic for another method, if desired. Every woman not lost to follow-up completed an interview (and queried again about side effects, her self-injection experience, and her desire to continue (or not) with self-injection), regardless of whether she had continued with self-injection.

### 2.2 Data collection and analysis

Structured, private, face-to-face interviews were conducted with each study participant. Interviews were conducted in French or the local language, Wolof (as preferred by participants), by female interviewers. Data were entered electronically on Android phones. The same study staff who enrolled women conducted the follow-up visits.

A sample size of 380 self-injectors was calculated to achieve a desired precision of 5 percentage points around the point estimate of 80% of women competent in self-injection at 3 months [95% CI=75–85%] and a precision of 5 percentage points around the point estimate of 80% of women reinjecting consistent with the schedule. We identified 80% as an appropriate competency estimate based on other studies that found observed or self-reported self-injection capability between 87% and 93% [8], [9], [11]. The sample size assumed 10% lost to follow-up and 20% discontinuation of the injectable.

End points for the primary objective of evaluating competency in self-injection, measured at the 3-month follow-up visit, were the percentage of women who demonstrated correct self-injection technique consistent with the observation checklist, and the percentage who reinjected on schedule, within 1 week of their reinjection date.

To qualify as competent, a participant had to successfully demonstrate five critical injection steps from the 19-step observation checklist (Appendix B) [15]. Omission of any of these five steps could lead to an ineffective injection. The steps were: (a) select an appropriate injection site, (b) mix the solution by shaking vigorously, (c) push the needle shield and port together to activate the device, (d) pinch the skin to form a ‘tent’, and (e) squeeze the reservoir slowly to inject the contraceptive. Women who discontinued the injectable (e.g., switched methods or stopped contraception in order to become pregnant) and women lost to follow-up at 3 months were not included in the denominator for the calculation of the primary outcomes.

Secondary end points on acceptability were measured at 3 months (for those discontinuing self-injection at that time) and 6 months (for those continuing with self-injection). Secondary end points on home storage and disposal practices were measured at 6 months, after the unsupervised self-injection.

Data analysis was performed using STATA version 14.1. 95% CIs for the primary end points were calculated using exact CIs for binomial proportions.

## 3 Results

### 3.1 Participant characteristics

A total of 380 participants were enrolled in the study, though two were later withdrawn due to undetected pregnancy (screening failure), leaving a sample of 378 participants at baseline. Table 1 summarizes the sample characteristics. Of particular note, over one third of participants had never been to school and over 80% were experienced injectable users, 34% DMPA-SC and 47% DMPA-IM.

**Table 1:** Baseline characteristics of participants.

	Percent or mean	n/N
Total		N=378
Mean age in years	27.7 (SD=6.3)	
Education		
None	36.0	136
Primary	34.9	132
Secondary/post-secondary	29.1	110
Marital status		
Married and cohabiting	78.3	296
Married, living apart	17.5	66
Unmarried	4.2	16
Mean parity	2.7 (SD=1.8)	
Contraceptive experience		
New user of family planning	11.6	44
New to injectable	7.9	30
Experienced DMPA-SC user	33.9	128
Experienced DMPA-IM user	46.6	176
Partner supports family planning use	86.0	325/378
Family support for family planning		
Very few/unknown	19.6	74
Some	25.7	97
Most	26.2	99
Nearly all	28.6	108
Community support for family planning		
Very few/unknown	20.1	76
Some	29.6	112
Most	24.3	92
Nearly all	25.9	98
Concerned about privacy at the clinic		
Not at all concerned	86.5	327
A little concerned	10.1	38
Very concerned	3.4	13
Motivation to try self-injection		
Very motivated	58.2	220
A little motivated	39.4	149
Not very motivated/uncertain	2.6	9
Common reasons to try self-injection		
Saves time/convenient	91.0	344
Saves money	46.8	177
Avoid missing work	22.0	83
Permits autonomy	14.6	55
More discreet	6.9	26
Level of anxiety about self-injection		
Not nervous	60.3	228
A little nervous	34.7	131
Very nervous	5.0	19
Mean number of practice attempts	3.2 (SD=.83)	
Paid for transport to travel	41.3	156/378
Mean travel expense (in $) if >0	0.40 (SD=0.54)	156
Missed work for clinic visit	29.6	112/378

### 3.2 Injection competence and adherence to reinjection schedule

Of these 378 women, 90% achieved competency at their first injection immediately post-training (Table 2). On average, women practiced 3.2 times on a prosthetic before self-injecting for the first time (Table 1). Of the 38 women judged not competent, half (19) declined to self-inject. For those who tried but failed, the most common reason for not reaching competence was failure to press the reservoir slowly (data not shown).

**Table 2:** Study participant competence and reinjection timing.

	%	n/N	95% CI
Competence at 1st injection⁎	89.9	340/378	86.5–92.8
Competence at 2nd injection (of those who self-injected)⁎⁎	86.5	268/310	82.1–90.1
Total competence (1st and 2nd injection)	79.5	268/337	74.8–83.7
Reinjection timing (2nd injection)		310	
2 weeks early (8–14 days)	0.3	1	
On time (+/− 1 week)	84.2	261	79.6–88.1
Up to 2 weeks late (8–14 days)	9.4	29	
Up to 3 weeks late (15–21 days)	3.2	10	
Up to 4 weeks late (22–28 days)	1.9	6	
More than 4 weeks late (29–35 days)	0.01	3	
Both competent and on time (2nd injection)	71.9	223/310	66.6–76.0
Reinjection timing (3rd injection)		283	
Did not recall reinjection date	2.1	6	
More than 4 weeks early (29–31 days)	0.7	2	
4 weeks early (22–28 days)	0	0	
3 weeks early (15–21 days)	0.3	1	
2 weeks early (8–14 days)	0.7	2	
On time (+/− 1 week)	90.8	257	86.8–93.9
Up to 2 weeks late (8–14 days)	2.5	7	
Up to 3 weeks late (15–21 days)	0.7	2	
Up to 4 weeks late (22–28 days)	0	0	
More than 4 weeks late (29–32 days)	2.1	6	

⁎ Denominator includes 19 women who did not self-inject the first injection, and 19 women who self-injected, but not competently.

⁎⁎ Between the 1st and 2nd injections, 5 women changed their mind about self-injection, 13 were lost to follow-up, 22 women switched to provider injection rather than self-inject and 28 women discontinued the injectable, leaving 310 women who went forward with the 2nd self-injection.

Of the 310 women who self-administered the second injection 3 months later, 87% injected competently without additional guidance, other than the client instruction booklet, which they were advised to reference. Ninety-eight percent reported that the booklet was “very easy” or “easy” to understand (data not shown). For the calculation of the total percent competent, 27 women who changed their mind about self-injection either immediately after the first injection or when due for the second injection were added to the denominator under the assumption that they declined to self-inject due to a lack of competence and/or confidence, resulting in a total competency finding of 80%. Women lost to follow-up between the enrollment visit and the clinic follow-up visit (n=13) and those who discontinued the injectable altogether (n=28) are not included in the calculation of total competency. For the second injection, not pressing the reservoir slowly and failing to push the needle shield and port together to activate the device were the most common causes of failure (data not shown). With respect to the timing of reinjection, 84% of women returned to the clinic on time (within 1 week of their scheduled injection date). Taking into consideration injection technique as well as timing, 72% of women were both on time for reinjection and administered the injection correctly.

Though the third injection was not observed, women were asked to provide the date that they reinjected. Of the 283 women who self-injected independently at home, 91% reported self-injecting within 1 week of their scheduled injection date (Table 2). Notably, nine women (3%) reported an injection date that fell outside of the DMPA reinjection window (−2/+4 weeks), either too early (*n*=3) or too late (*n*=6). Two women were lost to follow-up between the second and third injections.

### 3.3 Self-injection experience

**Fig. 1:**
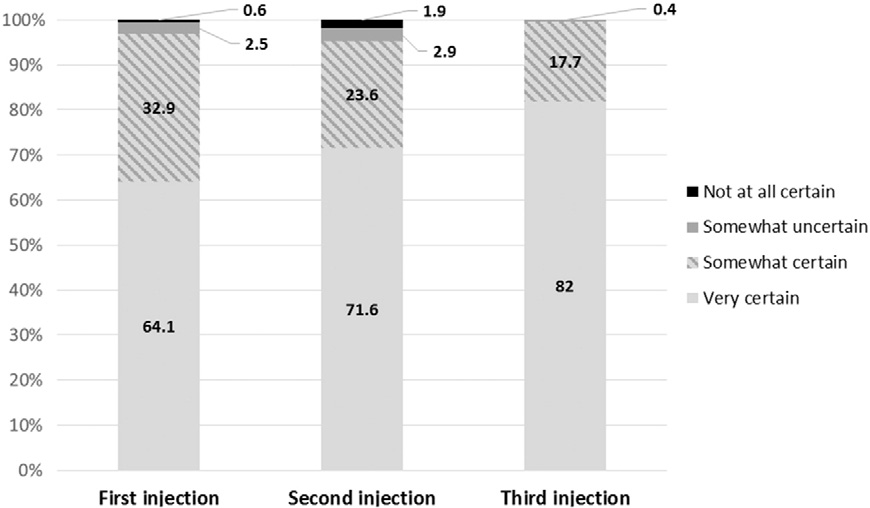
Confidence in self-injection administration.

In terms of subjective confidence, there is a pronounced increase in the percent that report feeling “very confident” that the injection was administered correctly, from 64% for the first self-injection to 82% for the third self-injection (Fig. 1). Consistent with this, the percent that indicated self-injection is ‘very easy to do’ increased from 64% to 72% between the first and third injections (Table 3). Among those who reported that self-injection was somewhat or very difficult, the most commonly reported challenges were inserting the needle and pressing the reservoir (first and third injections) and following the steps in the booklet (second injection). More than four out of five women self-injected in the thigh, 83–86%, depending on the injection (Table 3), and there was no difference in competency by injection site (data not shown). The most common strategies to recall the reinjection date were the calendar provided at enrollment (50%) and memorizing the date (33%). One in five women relied on friends, family or her husband/partner to recall the reinjection date.

**Table 3:** Self-injection experiences.

	First injection @ *N*=378		Second injection @ *N*=310		Third injection @ *N*=280	
Injection site⁎		358		289		
Thigh	86.3	309	86.2	249	83.4	236
Abdomen	13.7	49	13.8	40	15.5	44
Pain during injection						
Not painful	60.6	229	60.6	188	58.9	165
A little painful	39.2	148	38.4	119	41.1	115
Very painful	0.3	1	1.0	3	0.0	0
Pain after injection						
Not painful	84.7	320	83.2	258	76.4	214
A little painful	15.3	58	16.8	52	23.6	66
Very painful	0.0	0	0.0	0	0.0	0
Ease of giving injection						
Very easy	63.7	241	65.5	203	71.8	201
Somewhat easy	27.0	102	30.3	94	27.1	76
Somewhat difficult	7.4	28	3.6	11	1.1	3
Very difficult	1.9	7	0.7	2	0.0	0
Most difficult step⁎⁎		35		13		3
Inserting the needle	6.1	23	0.9	3	1.1	3
Pressing the reservoir	4.2	16	1.3	4	0.4	1
Remembering the steps	1.3	5	1.3	4	0.0	0
Using the booklet	0.8	3	1.6	5	0.0	0
Ease of remembering reinjection date	Not assessed		Not assessed			
Very easy					73.2	205
Somewhat easy					19.3	54
Somewhat difficult					6.1	17
Very difficult					1.4	4
Strategies to recall reinjection date	Not assessed		Not assessed			
Calendar provided at enrollment					50.4	141
Help from friend/family/husband					20.7	58
Help from provider					3.2	9
Programmed date into phone					2.1	6
Memorized the reinjection date					32.5	91

⁎ Questions about the injection site for the first and second injections were asked retrospectively, so results reflect a partial sample of those who were followed up subsequently.

⁎⁎ Only those who responded that the injection was somewhat or very difficult were asked to identify what was challenging.

### 3.4 Acceptability of self-injection

**Fig. 2:**
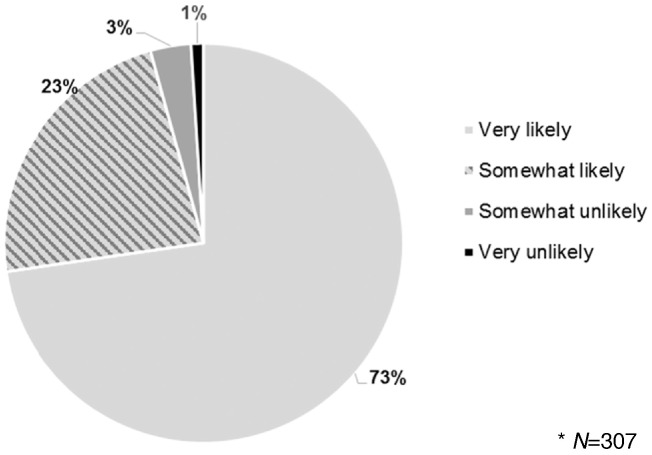
Likelihood of recommending self-injection to others*.

More than 9 (93%) in 10 women reported that they would like to do self-injection in the future if it were available, and nearly 3 (73%) in 4 women reported that they were “very likely” to recommend self-injection to others (and an additional 23% were “somewhat likely” to recommend it) (Fig. 2).

### 3.5 Safety

There were no pregnancies or serious adverse events. The most common adverse events were known side effects of DMPA: amenorrhea and other changes in menstrual bleeding patterns. After the first injection, 15 women reported experiencing an injection-site reaction (ISR) in the form of a dimple, bruise, blister, or nodule, and one woman sought advice or treatment. After the second injection, five women reported experiencing an ISR, of whom one sought treatment. All adverse events were mild to moderate in severity.

### 3.6 Storage and disposal

The vast majority of women (97%) reported that they were able to store the device at home securely, without discovery by children or others (Table 4). Nearly three-quarters (74%) stored the unit in an armoire or dresser.

**Table 4:** Device security, storage, and disposal at home, third injection.

	%	n/N
Device kept secure until use⁎	97.3	283/291
Storage location		291
Armoire/dresser	73.9	215
Suitcase	8.3	24
Handbag	5.5	16
Other location	12.4	36
Disposal practices⁎⁎		280
Pit latrine	48.9	137
Kept the device for the study nurse to collect	35.7	100
Returned device to clinic	10.7	30
Household or community garbage	3.2	9
Other	1.4	4
Placed in temporary safety container until disposal	48.6	136/280

⁎ All clients followed up were asked about storage, regardless of whether they had changed their mind about self-injection.

⁎⁎ Three individuals self-injected at the home visit and did not have the full range of disposal options (since the study nurse collected the spent device); they were therefore not asked about disposal practices.

In terms of disposal, about half of women (49%) disposed of the spent device in a pit latrine (Table 4). Most of the remaining women retained the device, knowing that study staff could retrieve it during the home visits, while 10% of women returned the device to the clinic for disposal. Only about half of women (49%) adhered to the instructions to place the spent device in an impermeable, empty household container with a lid — such as a Vaseline jar or water bottle — until it could be safely discarded.

## 4 Discussion

Two studies from sub-Saharan Africa, one in Senegal and one in Uganda, have now demonstrated that self-injection of DMPA-SC is feasible and acceptable for the vast majority of participants [13]. Of those women who actually self-injected 3 months after training, injection competence was similar in both countries (87% in Senegal vs. 90% in Uganda). In Senegal, a higher proportion of women opted not to self-administer their second injection relative to the proportion in Uganda, resulting in lower overall competence results in Senegal for the second injection.

Considering the similarities and differences in the two studies and contexts yields insights for potential self-injection program design. These findings align with previous studies that found higher acceptability of self-injection in Uganda than Senegal among DMPA-SC users [4], [5]. This difference may be attributable to higher overall prevalence of injectable contraceptive use in Uganda, as well as injectable contraception being available through community health workers in Uganda since 2010 [1], [16], [17]. Familiarity of communities with injectable contraception may be an enabling factor for introduction of self-injection. At the same time, the Senegal results, along with those from the Brazil Cyclofem® study, reinforce that some women will continue to opt for injections from providers and the importance of informed choice in contraceptive delivery [12], [18].

Underlying differences in the samples may explain the differences in outcomes observed. A much larger proportion of participants in Senegal had never attended school than in Uganda (about one-third compared with less than 10%). Strategies specifically designed to empower, train, and support women with limited education to self-inject can ensure that this service innovation is an option for all women; women in sub-Saharan Africa with the least education also have the lowest levels of contraceptive use [19].

Notably, women in Senegal returned to the clinic for their second self-injection, which was observed, while women in Uganda self-administered their second injection independently at home and later demonstrated their injection technique on a model. Women in Senegal may have encountered challenges returning to the clinic during the proscribed study window, reducing the proportion of women reinjecting “on time”. While we cannot know the extent to which competency differences between Uganda and Senegal is due to instrumentation, it is possible that performing injections under health worker supervision made participants feel less confident. Self-injection programs will need to be designed to offer sufficient support (including for product disposal) without compromising accessibility, autonomy, and discretion.

### 4.1 Study limitations

Requiring women to return to the clinic to demonstrate competence when due for the second injection may have reduced motivation to try self-injection — since the benefits were less immediate — and altered in unknown ways the profile of women for whom self-injection is appealing.

The conservative measurement of ‘on-time’ reinjection, with follow-up by a study nurse 7 days following the scheduled injection, precluded measurement of whether women would have self-injected within the established DMPA reinjection window (−2/+4 weeks). Nonetheless, the finding that a high percentage of women respected the very narrow reinjection window is encouraging for adherence to the more forgiving DMPA reinjection schedule.

Because of the study design — recruiting participants from among injectable clients — it was not possible to assess whether self-injection could attract new users to contraception. Future research should explore the appeal of self-injection among hard-to-reach populations (young women, new users, etc.) in order to evaluate its potential to increase contraceptive use.

## Footnotes

i While the reinjection window for DMPA is −2/+4 weeks, the study applied a more conservative 14-day grace period (−1/+1 week) for reinjection timing out of research ethics considerations, to ensure that study participants would not be at risk of pregnancy if unable to self-inject.
