# Supplementary material for: Evaluating the feasibility and acceptability of self-injection of subcutaneous depot medroxyprogesterone acetate (DMPA) in Senegal: a prospective cohort study
Source: Contraception. 2017 Sep;96(3):203–10. doi: 10.1016/j.contraception.2017.06.010 (PMC6381449; doi:10.1016/j.contraception.2017.06.010)
Supplement: Appendix A — SayanaPress_HSI_instructions_A6_English_2016-03-14. [file mmc1.pdf]

FOR CLIENTS

Sayana® Press
Self-injection Instructions

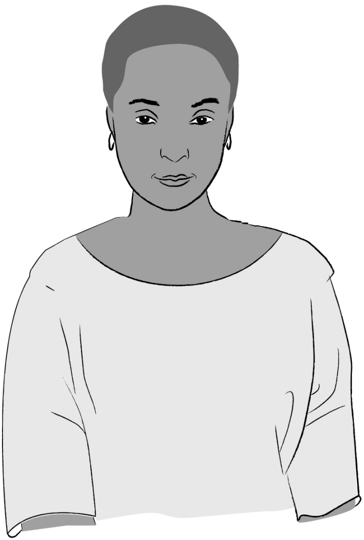

STEP 1: Wash your hands

- Use soap and water.

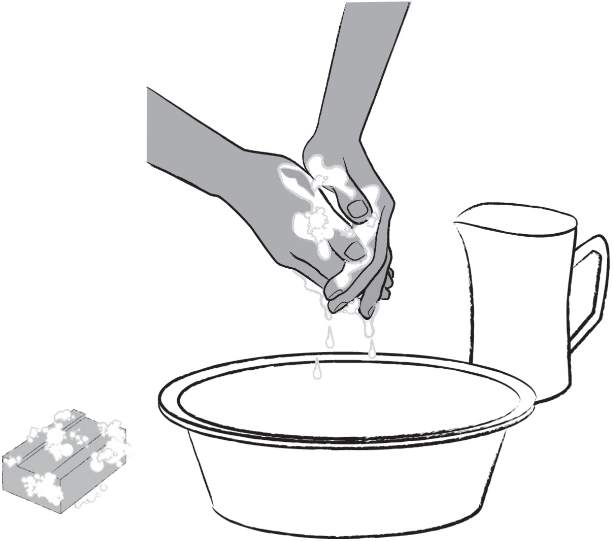

STEP 2: Select an injection site

- You can inject yourself with Sayana® Press in one of two places:

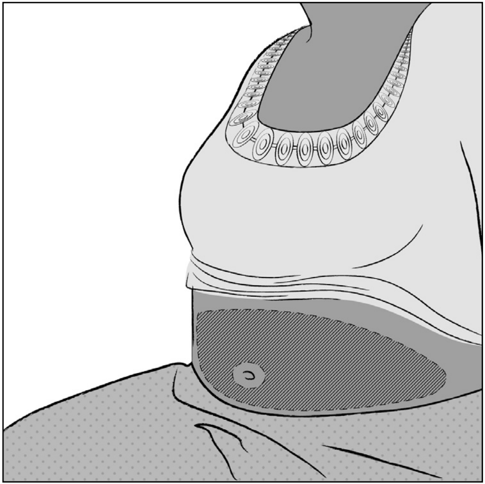

- The abdomen (NOT at the navel).

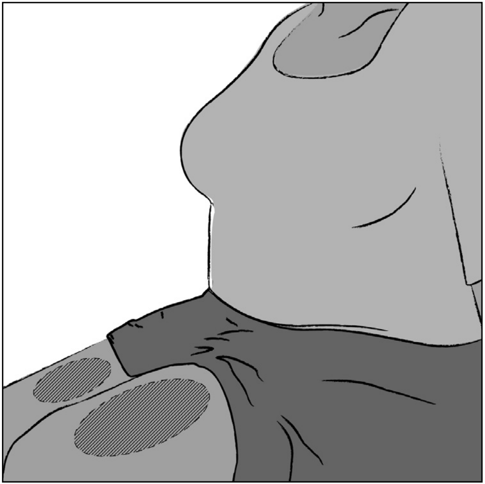

- The front of the thigh.

STEP 3: Open the pouch

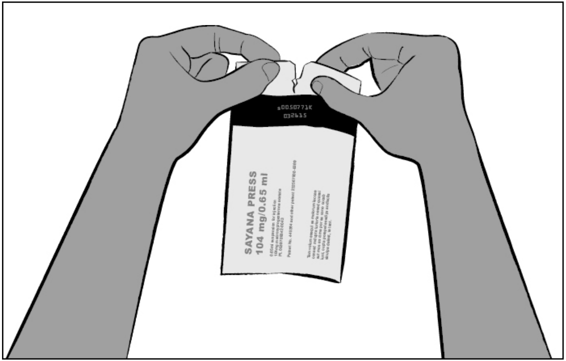

- Open the pouch and remove the device.
- Do not bend the device.

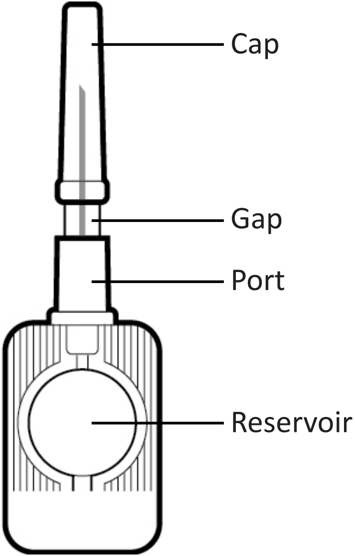

STEP 4: Mix the solution and check the device

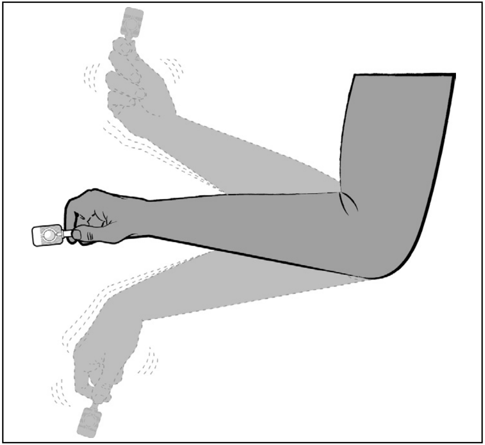

- Hold the device **by the port** and shake until mixed (about 30 seconds).

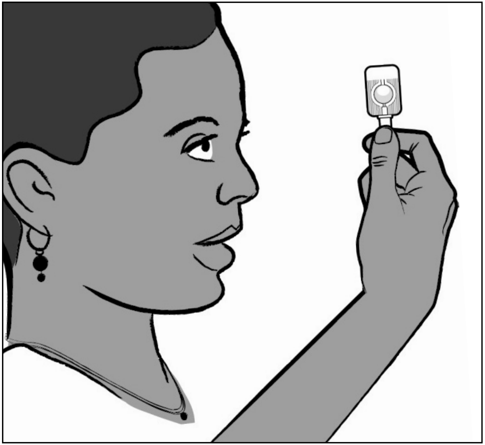

- Check to make sure there is no damage or leaking.
- If you do not inject right away, shake and mix again.

STEP 5: Activate the device by closing the gap

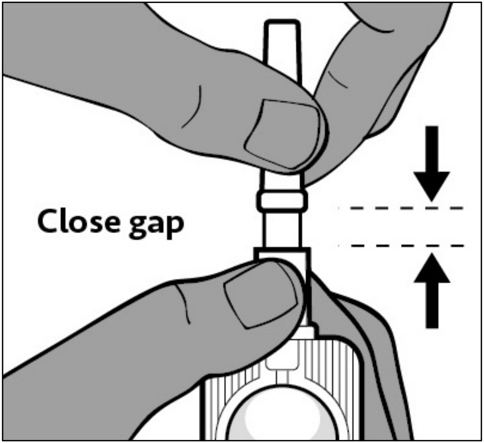

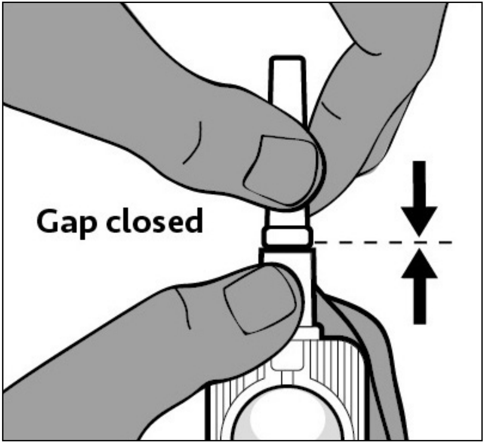

- Hold the device **by the port**.
- Point the needle upward during activation to prevent dripping.

- Push the cap firmly into the port.
- If the gap is not fully closed, you will not be able to press the reservoir during injection.

STEP 6: Remove the needle cap

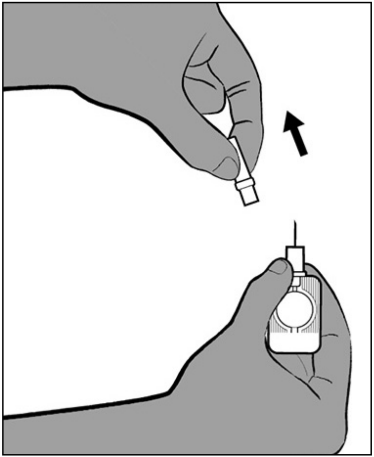

- Remove the needle cap.

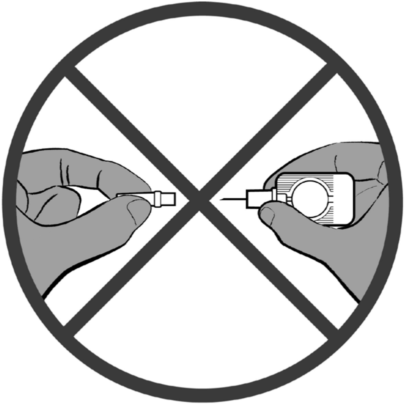

- Do not put the needle cap back on.

STEP 7: Gently pinch the skin at the injection site.

- This creates a “tent” for inserting the needle.

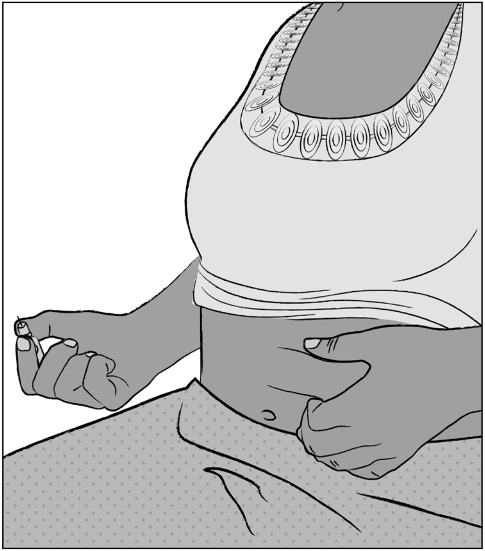

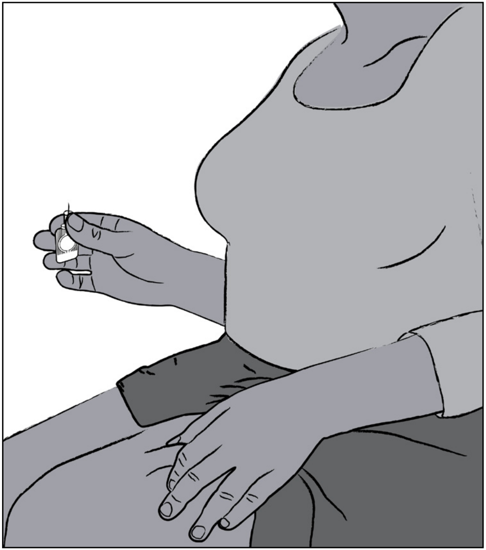

STEP 8: Insert the needle at a downward angle

- Continue to hold the device **by the port** and insert the needle straight into the skin at a downward angle.
- The port should touch the skin completely to ensure the needle is inserted at the correct depth.

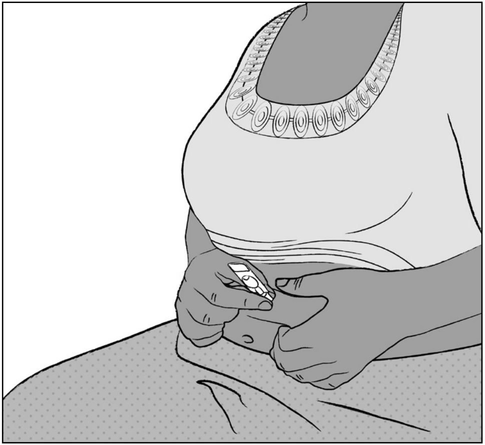

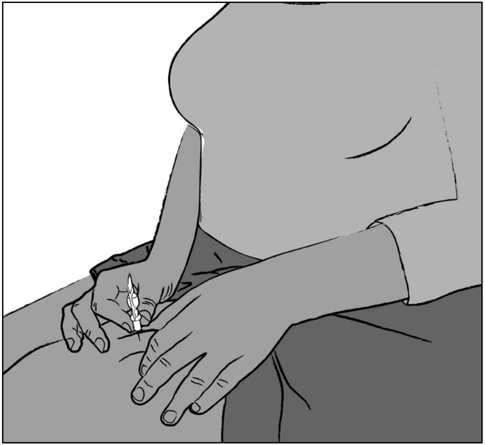

STEP 9: Press the reservoir

- Press the reservoir slowly for 5 to 7 seconds.
- It is OK if there is a little liquid left in the reservoir.

5 to 7 seconds

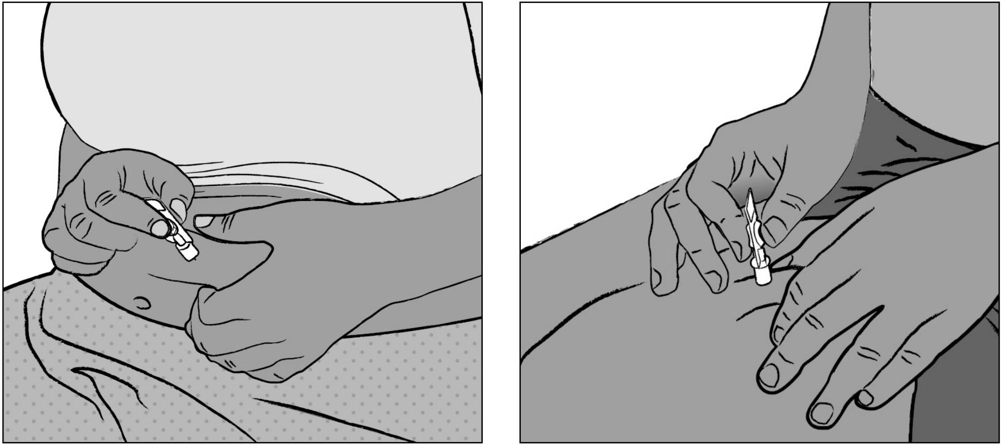

STEP 10: Remove the needle

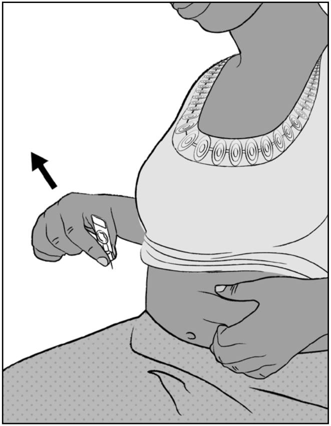

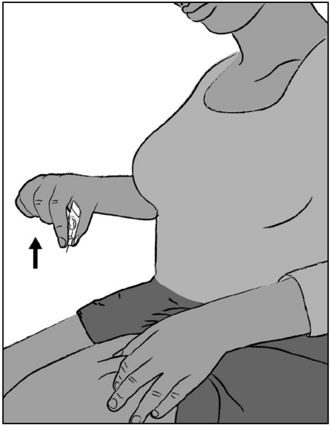

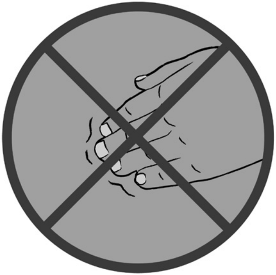

- Remove the needle from the injection site, then let go of the skin “tent.”
- Do not rub the injection site.

STEP 11: Discard the device

- Immediately discard the device in a puncture-proof container.
- Put on the container lid.

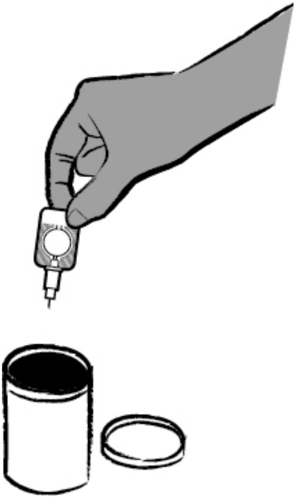

- Keep the container in a safe place away from children until:

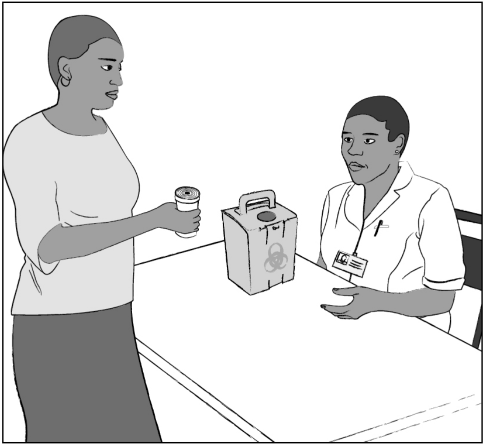

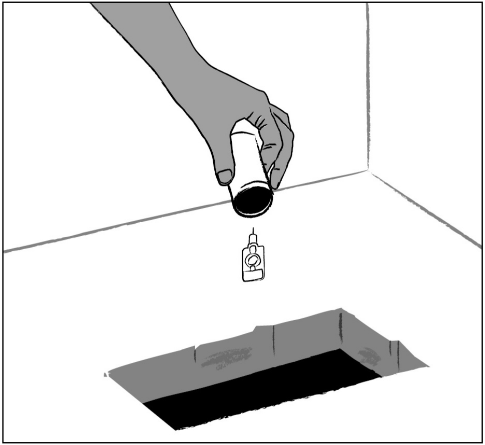

- You can give it to your health worker to be thrown out.

OR

- You can throw out the device in a pit latrine.

STEP 12: Plan for your next injection in 3 months

- Circle the date of your injection in your Sayana Press calendar.
- Also write that date on the next page (at right).
- Use your Sayana Press calendar to count 3 months to your next injection.
- Then circle your next injection date in your Sayana Press calendar.
- Also write that date on the next page (at right).

Example calendar

Month 1						
**Mon**	**Tues**	**Wed**	**Thu**	**Fri**	**Sat**	**Sun**
		1	(2)	3	4	5
6	7	8	9	10	11	12
13	14	15	16	17	18	19
20	21	22	23	24	25	26
27	28	29	30	31		

Month 2						
**Mon**	**Tues**	**Wed**	**Thu**	**Fri**	**Sat**	**Sun**
					1	2
3	4	5	6	7	8	9
10	11	12	13	14	15	16
17	18	19	20	21	22	23
24	25	26	27	28	29	30

Month 3						
**Mon**	**Tues**	**Wed**	**Thu**	**Fri**	**Sat**	**Sun**
1	2	3	4	5	6	7
8	9	10	11	12	13	14
15	16	17	18	19	20	21
22	23	24	25	26	27	28
29	30	31				

Month 4						
**Mon**	**Tues**	**Wed**	**Thu**	**Fri**	**Sat**	**Sun**
			1	(2)	3	4
5	6	7	8	9	10	11
12	13	14	15	16	17	18
19	20	21	22	23	24	25
26	27	28	29	30	31	

Your injection dates

Injection 1 ________________________________

Injection 2 ________________________________

Injection 3 ________________________________

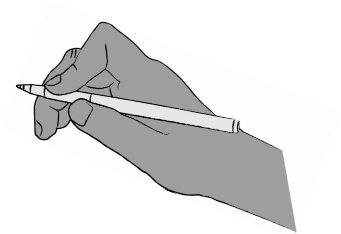

What if you miss your scheduled reinjection date?

If you are up to 1 month after your scheduled date:

- You can still give yourself an injection and be protected against pregnancy.
- Cross off the date you missed and circle your actual injection date.
- Then follow the **Step 12** instructions to plan your next injection date.

If you are more than 1 month after your scheduled injection date:

- Do not give yourself a Sayana Press injection.
- Contact your health worker.
- Use condoms or do not have sex until you speak with your health worker.

Example calendar

Month 1						
Mon	Tues	Wed	Thu	Fri	Sat	Sun
		1	2	3	4	5
6	7	8	9	10	11	12
13	14	15	16	17	18	19
20	21	22	23	24	25	26
27	28	29	30	31		

Month 2						
Mon	Tues	Wed	Thu	Fri	Sat	Sun
					1	2
3	4	5	6	7	8	9
10	11	12	13	14	15	16
17	18	19	20	21	22	23
24	25	26	27	28	29	30

Month 3						
Mon	Tues	Wed	Thu	Fri	Sat	Sun
1	2	3	4	5	6	7
8	9	10	11	12	13	14
15	16	17	18	19	20	21
22	23	24	25	26	27	28
29	30	31				

Month 4						
Mon	Tues	Wed	Thu	Fri	Sat	Sun
			1	2	3	4
5	6	7	8	9	10	11
12	13	14	15	16	17	18
19	20	[illegible]	22	23	24	25
26	27	28	29	30 X	31 X	X

OK to inject

Common Sayana Press side effects

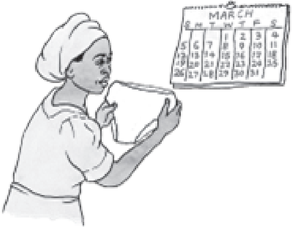

Common side effects can include the following and are not usually cause for concern:

- Lack of monthly bleeding.
- Heavy or irregular monthly bleeding.
- Headaches.
- Changes in mood or sex drive.
- Weight gain.
- Abdominal pain.

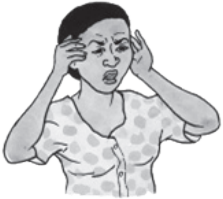

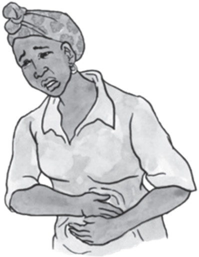

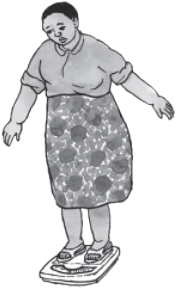

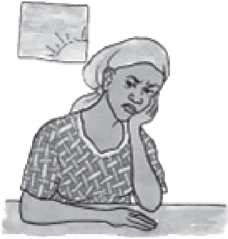

Other important information

Sayana Press does not protect against sexually transmitted infections such as HIV. Please use condoms in addition to Sayana Press to prevent against sexually transmitted infections.

Store Sayana Press in a safe place away from children or animals and extreme heat or cold.

If you experience pain or hardening at the injection site, or if you have questions about self-injection, your health, or side effects, contact a health worker:

Name ____

Phone ____

PATH
