# Supplementary material for: Evaluating the feasibility and acceptability of self-injection of subcutaneous depot medroxyprogesterone acetate (DMPA) in Senegal: a prospective cohort study
Source: Contraception. 2017 Sep;96(3):203–10. doi: 10.1016/j.contraception.2017.06.010 (PMC6381449; doi:10.1016/j.contraception.2017.06.010)
Supplement: Appendix B — Observation Checklist. [file mmc2.docx]

## Observation checklist for Sayana Press injection practice

Woman number |___|___|___|___|___| Health center number |____|____|

Interviewer code |___|___|___| Date: DD/MM/YY |____|____|____|

**Use this checklist to evaluate whether a study participant injects correctly, according to objective performance standards. Give the participant the Self-injection Booklet to use as a guide. The first five observations are for practice on a model. The last observation is for the actual self-injection (SI). For each practice attempt, tick the box (√) for each step that is done correctly. If a step is done incorrectly or skipped, write an X in the corresponding box.**

| **Steps** | Observations  √ or X | | | | | |
| --- | --- | --- | --- | --- | --- | --- |
|  | 1 | 2 | 3 | 4 | 5 | SI |
| 1. Washes hands (may be mimicked for practice on models) |  |  |  |  |  |  |
| 1. **Selects an injection site (thigh or abdomen) and cleans if needed.** |  |  |  |  |  |  |
| 1. Opens the Sayana Press pouch by tearing the notch. |  |  |  |  |  |  |
| 1. Holds the device by the port while mixing. |  |  |  |  |  |  |
| 1. **Mixes the liquid by shaking the device vigorously (about 30 seconds).** |  |  |  |  |  |  |
| 1. Checks to make sure the liquid is mixed and there is no damage to the device. |  |  |  |  |  |  |
| 1. Holds the device with the needle pointing upward during activation. |  |  |  |  |  |  |
| 1. Holds the device by the port while activating. |  |  |  |  |  |  |
| 1. **Pushes the needle cap and port together to activate the device.** |  |  |  |  |  |  |
| 1. **Pinches the “skin” at the injection site to form a tent.** |  |  |  |  |  |  |
| 1. Holds the port of the device while inserting the needle. |  |  |  |  |  |  |
| 1. Inserts the needle into the tent of skin between the thumb and forefinger. |  |  |  |  |  |  |
| 1. Inserts the needle at a downward angle. |  |  |  |  |  |  |
| 1. Inserts the needle completely so that the port is in full contact with the skin. |  |  |  |  |  |  |
| 1. Moves fingers from the port to the reservoir while still pinching the skin. |  |  |  |  |  |  |
| 1. **Presses the reservoir slowly to inject — taking about 5–7 seconds.** |  |  |  |  |  |  |
| 1. Removes the device from the injection site while still pinching skin. |  |  |  |  |  |  |
| 1. Does not rub the injection site. |  |  |  |  |  |  |
| 1. Places the used device immediately into a sharps disposal container without replacing the needle cap. |  |  |  |  |  |  |
| **Total the number of √ marks (steps completed correctly) for each practice attempt and enter the total at the bottom.**  **Circle any of the critical steps (bold) that the woman does NOT do correctly.** |  |  |  |  |  |  |
|  |  |  |  |  |  |  |
| In your clinical judgment, has this individual demonstrated sufficient competence to self-inject independently AND did the woman do all the 5 critical steps (bold) correctly? YES NO | | | | | | |
